# Empirical examination of the indicator ‘pediatric gastroenteritis hospitalization rate’ based on administrative hospital data in Italy

**DOI:** 10.1186/1824-7288-40-14

**Published:** 2014-02-11

**Authors:** Jacopo Lenzi, Lorenza Luciano, Kathryn Mack McDonald, Simona Rosa, Gianfranco Damiani, Giovanni Corsello, Maria Pia Fantini

**Affiliations:** 1 Department of Biomedical and Neuromotor Sciences, Alma Mater Studiorum – University of Bologna, Bologna, Italy; 2 Center for Health Policy/Center for Primary Care and Outcomes Research, Stanford University, Stanford, USA; 3 Institute of Hygiene, Catholic University of the Sacred Heart, Rome, Italy; 4 Department of Sciences for Health Promotion and Mother and Child Care, University of Palermo, Palermo, Italy

**Keywords:** Health services research, Quality of care, Quality indicators, Pediatrics, Gastroenteritis

## Abstract

**Background:**

Awareness of the importance of strengthening investments in child health and monitoring the quality of services in the pediatric field is increasing. The Pediatric Quality Indicators developed by the US Agency for Healthcare Research and Quality (AHRQ), use hospital administrative data to identify admissions that could be avoided through high-quality outpatient care. Building on this approach, the purpose of this study is to perform an empirical examination of the ‘pediatric gastroenteritis admission rate’ indicator in Italy, under the assumption that lower admission rates are associated with better management at the primary care level and with overall better quality of care for children.

**Methods:**

Following the AHRQ process for evaluating quality indicators, we examined age exclusion/inclusion criteria, selection of diagnostic codes, hospitalization type, and methodological issues for the ‘pediatric gastroenteritis admission rate’. The regional variability of hospitalizations was analyzed for Italian children aged 0–17 years discharged between January 1, 2009 and December 31, 2011. We considered hospitalizations for the following diagnoses: non-bacterial gastroenteritis, bacterial gastroenteritis and dehydration (along with a secondary diagnosis of gastroenteritis). The data source was the hospital discharge records database. All rates were stratified by age.

**Results:**

In the study period, there were 61,130 pediatric hospitalizations for non-bacterial gastroenteritis, 5,940 for bacterial gastroenteritis, and 38,820 for dehydration. In <1-year group, the relative risk of hospitalization for non-bacterial gastroenteritis was 24 times higher than in adolescents, then it dropped to 14.5 in 1- to 4-year-olds and to 3.2 in 5- to 9-year-olds. At the national level, the percentage of admissions for bacterial gastroenteritis was small compared with non-bacterial, while including admissions for dehydration revealed a significant variability in diagnostic coding among regions that affected the regional performance of the indicator.

**Conclusions:**

For broadest application, we propose a ‘pediatric gastroenteritis admission rate’ that consists of including bacterial gastroenteritis and dehydration diagnoses in the numerator, as well as infants aged <3 months. We also suggest adjusting for age and including day hospital admissions. Future evaluation by a clinical panel at the national level might be helpful to determine appropriate application for such measures, and make recommendations to policy makers.

## Background

Thorough understanding and international comparisons of the health needs of children and the ways in which health systems address these needs require appropriate data [1]. Moreover, children are often an afterthought when health information systems are created: missing data, different methodology/patterns in developing measures for quality assessment (e.g., different coding habits for diagnosis inclusion in hospitalization rates) often prevent indicators to be transferable between countries.

Simply applying indicators to younger age ranges is insufficient because specific characteristics distinguish children from being simply ‘little adults’ [2,3]. Optimally, indicators should be adapted to age groups and contexts of care to enable comparative evaluation of the quality of care provided by health services, particularly when health systems are different [4].

Although some European projects have provided sets of indicators for children for primary and secondary care evaluation (Child Health Indicators of Life and Development [CHILD] project, Organization for Economic Co-operation and Development [OECD] pediatric indicators) [5], few research studies focus on proper methodology to develop such indicators and adapt them to different contexts.

A systematic attempt to measure the quality of child health services has been made in the US with the Pediatric Quality Indicators (PDIs) developed by the Agency of Healthcare Research and Quality (AHRQ) in collaboration with Stanford University and the University of California [6]. The PDIs are a set of measures based on hospital discharge data that provide a perspective on the quality of pediatric healthcare; they focus on iatrogenic events and potentially preventable complications for patients treated in hospitals. In particular, the PDIs released in 2006 included several indicators to identify potentially avoidable hospitalizations for ‘ambulatory care-sensitive’ conditions (ACSCs). These conditions include asthma, gastroenteritis, short-term diabetes complications, perforated appendix, and urinary tract infections [6,7].

The underlying concept is that timely and effective ambulatory care ensured by an integrated comprehensive system of providers and services with a preventive orientation, may avoid hospitalizations due to the mentioned conditions. According to the AHRQ, the development process of such indicators requires an empirical analysis in order to establish their validity and reliability, detect bias and design appropriate risk adjustment models before submitting candidate indicators to clinical panelists.

These quality indicators were the first set of measures developed exclusively for children using administrative databases and, to our knowledge, they have not been extensively used in Italy, even if some of them have been recently proposed by the National Outcomes Program (PNE, *Programma Nazionale Esiti*) [8]. Considering all pediatric hospitalizations due to ACSCs, dehydration/gastroenteritis is the second most common after asthma [9]. Gastroenteritis, in fact, is the most frequent reason for unscheduled visits to pediatricians after respiratory infections, and timely and effective care such as oral rehydration therapy may reduce the need of hospitalization [10].

Viruses remain by far the most common cause of acute gastroenteritis in children, in both industrialized and developing countries, though other bacterial and parasitic enteropathogens can be involved in the onset of the disease [11]. Rotavirus is the leading cause of severe acute gastroenteritis (vomiting and severe diarrhea) among children worldwide. Rotavirus vaccine was found to prevent almost all severe rotavirus infections (85% to 98%) and 74% to 87% of all rotavirus episodes. For these reasons, the Center for Disease Control and Prevention (CDC) currently recommends the use of the two different rotavirus oral vaccines licensed for infants in the US [12].

The introduction of the vaccination was found to be associated with a dramatic reduction in hospital admissions for acute gastroenteritis among US children during the 2008 rotavirus season [13]. Vaccination against the rotavirus has been recently introduced in Italy, even if not yet on a national scale, within the National Vaccination Plan 2012–14. Further investigation on the effectiveness of vaccination in reducing hospital admissions linked to dehydration/gastroenteritis in Italy would be helpful to orient national and regional policies towards increased adherence to the vaccination campaign [14]. This proves again the need to provide reliable and suitable indicators for monitoring activities and comparative evaluations in our healthcare setting.

The aim of the current study was to perform an empirical examination of the ‘pediatric gastroenteritis admission rate’ indicator in Italy.

## Methods

The key elements of ‘pediatric gastroenteritis admission rate’ indicator were examined through empirical analyses, as proposed by the AHRQ. These included:

(1) Diagnostic codes selection;

(2) Age exclusion/inclusion criteria;

(3) Hospitalization type: daytime hospital care (‘day hospital admissions’)^a^*vs.* ordinary hospitalizations;

(4) Age adjustment *vs.* stratification by age for comparative purposes.

The data source was the Italian hospital discharge records (HDRs) database, and Census data for the indicator’s area-level denominator. The HDR database includes demographic characteristics, admission and discharge dates, admission referral source, discharge status, principal diagnosis, up to five secondary diagnoses, and up to six interventions. HDRs are sent by all public and private hospitals to their own region, and every six months from the region to the Ministry of Health. Since 1995, all information stored in regional HDR databases has been consistent and harmonized at the national level. The HDR-DRG (Diagnosis Related Group) system is systematically used to allocate funds to hospitals and to monitor quality of care and outcomes.

The population of interest included all patients aged 0–17 years discharged between January 1, 2009 and December 31, 2011 with a primary diagnosis of non-bacterial gastroenteritis (ICD-9-CM codes: 008.6x, 008.8, 009.x, 558.9) or bacterial gastroenteritis (003.0, 003.8, 003.9, 004.x, 008.0x–008.5)^b^. We also analyzed pediatric hospital admissions for dehydration (276.5x) as primary diagnosis, when the secondary diagnosis was gastroenteritis (either bacterial or non-bacterial). A detailed list of ICD-9-CM diagnosis codes included in the analyses is provided as Additional file 1.

Records were excluded from the analysis if the following criteria were met:

(1) Major Diagnostic Category code 14 (pregnancy, childbirth and puerperium);

(2) Transfers from other hospitals;

(3) A diagnostic code of gastrointestinal abnormalities (538 [gastrointestinal ulcerative mucositis], 555.x [regional enteritis], 556.x [ulcerative enterocolitis], 558.1–558.3 [toxic/allergic gastroenteritis and colitis, or due to radiation], 579.x [intestinal malabsorption]).

Pediatric admission rates were calculated as the average annual number of hospitalizations over the population aged 0–17 years per 1,000 inhabitants. Specific hospitalization rates were calculated for the following age groups: <1, 1, 2–4, 5–9, 10–14, 15–17 years. Age-adjusted rates were computed using the Italian population aged 0–17 years on January 1, 2002 as the standard population. The regional variability of hospitalization rates was examined through caterpillar plots and stacked bar charts. All analyses were performed using Stata software, version 13 (StataCorp. 2013. *Stata Statistical Software: Release 13.* College Station, TX: StataCorp LP).

The study was carried out in compliance with the Italian law on privacy (Art. 20–21, DL 196/2003). Data were encrypted with a unique identifier assigned to each patient. This identifier does not allow tracing the patient’s identity and other sensitive data. When encrypted administrative data are used to inform healthcare planning activities, studies are exempt from notification to the Ethics Committee and no specific written consent is needed to use patient information stored in the hospital databases.

## Results

Over the study period (2009–11) the number of pediatric hospitalizations (0–17 years) was 61,130 (average annual rate: 1.99 per 1,000) for non-bacterial gastroenteritis, 5,940 (0.19 per 1,000) for bacterial gastroenteritis, and 38,820 (1.27 per 1,000) for dehydration. Table 1 shows the hospital admission rates stratified by age group and the relative risks in children at different ages compared with adolescents. In <1-year group, the relative risk of hospitalization for non-bacterial gastroenteritis was 24 times higher than in adolescents, then it dropped to 14.5 in 1- to 4-year-olds and to 3.2 in 5- to 9-year-olds. Results were similar for bacterial gastroenteritis and dehydration: compared with adolescents, infants and preschool children had a higher risk of hospitalization for these conditions (Table 1).

**Table 1:** Pediatric hospital admissions in Italy by primary diagnosis and age group (period 2009–11)

**Age group**	**N**	%^*^	**Average annual rate (per 1,000)**	**RR (95% CI)**
***Non-bacterial gastroenteritis***				
<1-year^†^	13,492	3.5	7.99	24.2 (23.0–25.5)
1–4 years	32,735	6.0	4.79	14.5 (13.8–15.2)
5–9 years	8,959	2.4	1.06	3.2 (3.0–3.4)
10–14 years	4,216	1.2	0.50	1.5 (1.4–1.6)
15–17 years	1,728	0.7	0.33	Ref.
*Total*	*61,130*	*3.3*	*1.99*	
***Bacterial gastroenteritis***				
<1-year^‡^	750	0.2	0.44	8.8 (7.5–10.4)
1–4 years	2,949	0.5	0.43	8.6 (7.4–9.9)
5–9 years	1,352	0.4	0.16	3.2 (2.9–4.0)
10–14 years	653	0.2	0.08	1.6 (1.5–2.0)
15–17 years	236	0.1	0.05	Ref.
*Total*	*5,940*	*0.3*	*0.19*	
***Dehydration***				
<1-year^§^	6,047	1.6	3.58	59.7 (54.3–68.3)
1–4 years	23,389	4.3	3.42	57.0 (52.0–65.1)
5–9 years	6,646	1.8	0.78	13.0 (11.9–14.9)
10–14 years	2,430	0.7	0.29	4.8 (4.4–5.5)
15–17 years	308	0.1	0.06	Ref.
*Total*	*38,820*	*2.1*	*1.27*	

^*^Percentage over total pediatric hospitalizations in Italy (except Major Diagnostic Category codes 14 [pregnancy, childbirth and puerperium] and 15 [newborns and other neonates]).

^†^In <1-year group, 2,425 children (18.0%) were aged less than 3 months.

^‡^One hundred fifty-two children (20.3%) were aged less than 3 months.

^§^Six hundred fifty-eight children (10.9%) were aged less than 3 months.

RR, relative risk; 95% CI, 95% confidence interval.

*Data source:* Ministry of Health.

**Figure 1:**
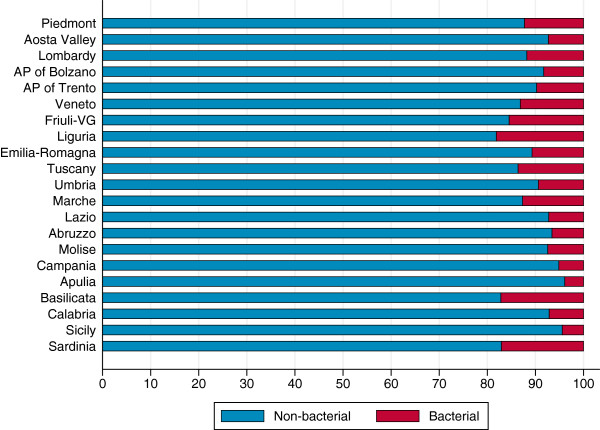
**Percentages of hospital admissions for non-bacterial and bacterial gastroenteritis by region (0–17 years).** AP, autonomous province; VG, Venezia Giulia. *Data source:* Ministry of Health.

**Figure 2:**
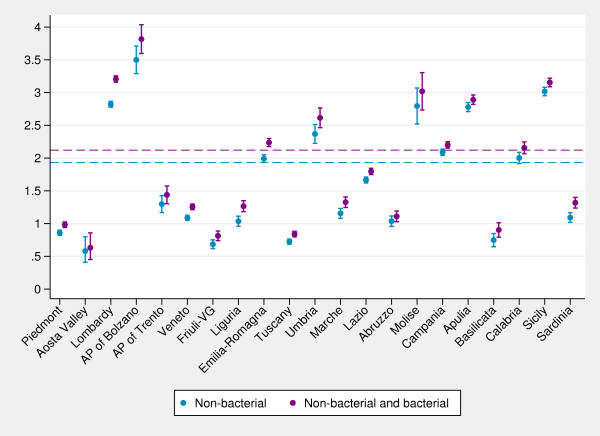
**Caterpillar plot of age-standardized regional admission rates (per 1,000) for non-bacterial gastroenteritis and for non-bacterial and bacterial gastroenteritis combined (0–17 years). ***Note:* Dashed lines, national averages. *Data source:* Ministry of Health.

The percentage of hospitalizations for non-bacterial and bacterial gastroenteritis by region is presented in Figure 1. As expected, we found a small percentage of bacterial gastroenteritis hospital admissions at the national level (8.9%), with small variability among regions. As a result, the inclusion of bacterial gastroenteritis diagnoses barely affects the regional performance of the indicator compared with the national average (Figure 2).

**Figure 3:**
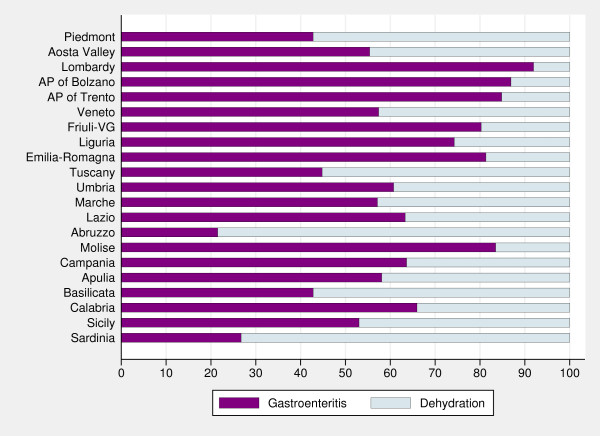
**Percentages of hospital admissions for gastroenteritis and dehydration by region (0–17 years). ***Note:* Gastroenteritis comprises both non-bacterial and bacterial diagnoses. *Data source:* Ministry of Health.

**Figure 4:**
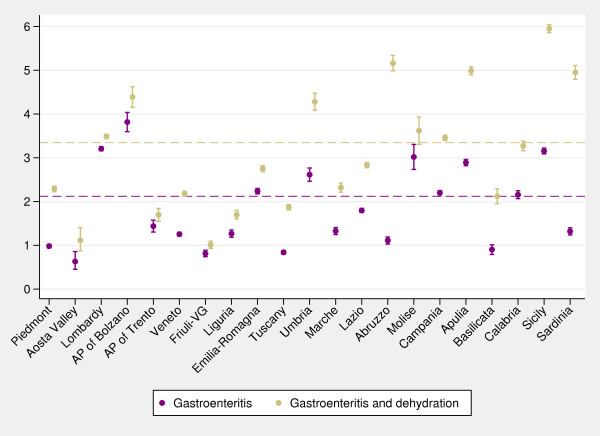
**Caterpillar plot of regional age-standardized admission rates (per 1,000) for gastroenteritis and for gastroenteritis and dehydration combined (0–17 years). ***Note:* Dashed lines, national averages. Gastroenteritis comprises both non-bacterial and bacterial diagnoses. *Data source:* Ministry of Health.

Stacked bar charts showing the percentages of admissions for dehydration and gastroenteritis (either bacterial or non-bacterial) (Figure 3), show huge variability in diagnostic coding among regions: in five regions more than 50% of hospitalizations were coded as primarily due to dehydration, whereas in regions such as Lombardy and the two autonomous provinces of Trento and Bolzano approximately 10% of hospital admissions received a primary diagnosis of dehydration. The inclusion of dehydration diagnostic code affects the regional performance of the indicator compared with the national average (Figure 4). In particular, if we consider hospitalizations for gastroenteritis and dehydration, the national rate strongly increases, as well as the variability among regions; moreover, in two regions (Abruzzo and Sardinia) rates of gastroenteritis-and-dehydration together are even five times higher than rates of gastroenteritis coded as primary diagnosis.

Since the AHRQ excludes infants aged <3 months from the indicator, we investigated the proportion of dehydration and gastroenteritis and its variability among regions for two age groups in the first year of life: <3- and 3- to 11-month-olds. At the national level, we found a lower proportion of dehydration in <3-months group compared with 3–11 months group (20.3% *vs.* 31.6%, respectively), and considerable variability among regions in both groups (see Additional file 2: Figure S1 and Additional file 3: Figure S2). However, regional pattern of diagnostic coding was similar between the two age groups considered: regions (Piedmont, Tuscany, Umbria, Abruzzo, Basilicata and Sardinia) with the highest proportions of dehydration hospital admissions in <3-months group (see Additional file 2: Figure S1) had the highest proportions of dehydration admissions also in 3–11 months group (see Additional file 3: Figure S2).

**Figure 5:**
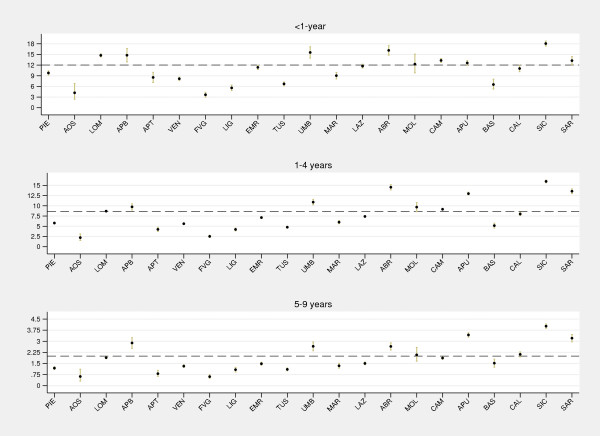
**Caterpillar plot of regional admission rates (per 1,000) for gastroenteritis and dehydration (<1-, 1- to 4-, and 5- to 9-year-olds).** PIE, Piedmont; AOS, Aosta Valley; LOM, Lombardy; APB, Autonomous province of Bolzano; APT, Autonomous province of Trento; VEN, Veneto; FVG, Friuli-Venezia Giulia; LIG, Liguria; EMR, Emilia-Romagna; TUS, Tuscany; UMB, Umbria; MAR, Marche; LAZ, Lazio; ABR, Abruzzo; MOL, Molise; CAM, Campania; APU, Apulia; BAS, Basilicata; CAL, Calabria; SIC, Sicily; SAR, Sardinia. *Note:* Dashed lines, national averages. Gastroenteritis comprises both non-bacterial and bacterial diagnoses. *Data source:* Ministry of Health.

Furthermore, we explored the need for age-stratification for regional comparative analyses. Caterpillar plots of age-specific gastroenteritis-and-dehydration admission rates (Figure 5) revealed similar regional patterns of the indicator across the age groups considered (<1-, 1- to 4-, and 5- to 9-year-olds), with a risk of hospital admission significantly higher in younger age groups. A similar regional pattern is also confirmed for children aged 10 years or more (figure not shown). Thus stratification by age is unnecessary, as the age pattern of hospital admissions does not vary across regions.

Lastly, we explored the effect of type of hospitalization by including day hospital admissions in the analysis. In Italy, only 2% of all gastroenteritis-and-dehydration hospitalizations were day hospital admissions, with low rates for all regions with some variability (range: 0.4% in Lombardy to 7.4% in Basilicata).

## Discussion

The current study aimed to add a second candidate indicator – after pediatric asthma admission rate – to the list of ACSCs suitable to be used in quality evaluation of primary care and preventive services in Italy [15]. While asthma is the prototype of a chronic condition, gastroenteritis represents the most common acute disorder in childhood, for which good primary care management can avoid hospitalizations. Gastroenteritis admission rates for newborns and younger children were much higher than for adolescents (the same was found for asthma) [15], and this clinically expected variation by age provides face validity evidence for the indicator.

As to the occurrence of non-bacterial and bacterial gastroenteritis, we found that the latter cases are infrequent and marginally affect the value of the indicator. The AHRQ Pediatric Panel Review excluded bacterial gastroenteritis diagnoses from the calculation of the indicator, as they may require hospitalization despite high-quality outpatient care [3]. With regard to this point we think that, despite the low prevalence of this condition, a clinical panel should decide whether to include or exclude this diagnosis on the basis of the pediatric primary care organization in Italy, where pediatricians act as primary care physicians in charge of providing also home visits, when needed. However, should this indicator be used to evaluate the impact of rotavirus vaccination campaigns, we recommend excluding all bacterial gastroenteritis diagnoses from the numerator.

Considering dehydration admissions (primary diagnosis along with a secondary diagnosis of gastroenteritis), our findings suggest a particularly frequent use of this code in specific regions. It is unlikely that this is due to different systems of hospital reimbursement, because gastroenteritis and dehydration have similar severity (mean length of stay: 3.2 and 3.4 days, respectively), and the two DRGs to which they are mainly linked (184 [esophagitis and gastroenteritis <18 years] and 298 [symptoms concerning nutrition and metabolism <18 years]) have very similar national relative weights (0.30 and 0.27) (ministerial decree 12/18/2008, link: http://www.sioechcf.it/allegati/DM_18.12.2008-Nuovi_ICD-9-CM_e_DRG.pdf). Even these differences do not seem to be related to variations in regional prevalence, because hospital admission rates do not have a clear North–south pattern consistent with climate differences. We thus argue that variability in admission rates may be explained by differences in coding habits and pediatric primary care practice, and that excluding dehydration codes, as currently proposed by the Italian PNE, would result in missing many cases with gastroenteritis.

Moreover, we examined exclusion of admissions during the first 3 months of life from the indicator, as proposed by the AHRQ. Regional coding habits proved to be similar and consistent across the two age groups considered (<3-months and 3–11 months), thus exclusion of the newborns aged <3 months does not affect the indicator. The indicator proposed by the PNE considers the pediatric population aged 0 to 17 years.

Our empirical analysis confirms the need for age-adjustment when comparative evaluations are performed. In fact, the risk of hospital admission is significantly higher in younger age groups; noteworthy, it is not necessary to stratify by age group, as age is not an effect modifier of regional performance.

Lastly, we found a very low proportion of day hospital admissions for gastroenteritis and dehydration at the national level, but some variability among regions. Thus, we argue that including day hospital admissions in the numerator would be worthwhile, so as to capture all potentially avoidable hospital admissions for gastroenteritis and dehydration, even shorter ones.

## Conclusions

As for another ACSC indicator for the pediatric age (i.e., asthma hospital admission rate), it is recommended to adapt the ‘pediatric gastroenteritis admission rate’ to the Italian healthcare setting. Our empirical analysis highlights some key points to be discussed by a structured clinical panel review involving specialists from the pediatric field at the national level.

Acute gastroenteritis hospitalizations might be particularly important also to evaluate the impact of rotavirus vaccination campaigns. As already mentioned, since pediatric acute gastroenteritis in industrialized countries is mainly caused by rotavirus, rotavirus vaccines will be introduced in upcoming infant vaccination plans to reduce avoidable hospitalizations and complications due to this common acute pediatric condition. Following the example of a recent US vaccination campaign introduction, where RV5 vaccine proved to dramatically reduce the hospitalization rates due to pediatric gastroenteritis [13], we submit that measures based on administrative data should be routinely collected and made systematically available to monitor vaccination effectiveness and cost-effectiveness. Conceived for this purpose, the HDRs database might become part of a future surveillance system to monitor adherence and coverage of vaccination, and pediatric ACSCs might be an important instrument to evaluate the quality of children healthcare.

## Endnotes

^a^In Italy, daytime hospital care (day hospital) consists in a planned one-day admission to the hospital without overnight stay, to perform diagnostic procedures and/or surgical, therapeutic or rehabilitative care. These diagnostic and therapeutic procedures require specific instrumental techniques, are carried out by multidisciplinary staff and are more complex than those run on an outpatient basis.

^b^Bacterial gastroenteritis diagnoses were included in the analysis because we assume that they are potentially preventable in Italy, where family pediatricians are trained specialists who work at the primary-care level providing ambulatory and home care.

## Abbreviations

CHILD: Child Health Indicators of Life and Development; OECD: Organization for Economic Co-operation and Development; US: United States; AHRQ: Agency for Healthcare Research and Quality; PDI: Pediatric Quality Indicator; CDC: Center for Disease Control and Prevention; ACSC: Ambulatory Care Sensitive Condition; PNE: Programma Nazionale Esiti [National Outcomes Program]; HDR: Hospital Discharge Records; ICD-9-CM: International Classification of Diseases (Clinical Modification, Ninth Revision); RR: Relative Risk; AP: Autonomous province; VG: Venezia Giulia; PIE: Piedmont; AOS: Aosta Valley; LOM: Lombardy; APB: Autonomous province of Bolzano; APT: Autonomous province of Trento; VEN: Veneto; FVG: Friuli-Venezia Giulia; LIG: Liguria; EMR: Emilia-Romagna; TUS: Tuscany; UMB: Umbria; MAR: Marche; LAZ: Lazio; ABR: Abruzzo; MOL: Molise; CAM: Campania; APU: Apulia; BAS: Basilicata; CAL: Calabria; SIC: Sicily; SAR: Sardinia.

## Competing interests

The authors declare that they have no competing interests.

## Authors’ contribution

JL conceived the statistical methodology, performed the statistical analysis and drafted the manuscript; LL contributed to the conception of this paper, conceived the study design and drafted the manuscript; KMM participated in the study design and helped to draft the manuscript; SR took responsibility for the integrity of the data and performed the statistical analysis; GD provided data sources and participated in the study design; GC critically revised the draft and contributed to the final writing of the paper; MPF contributed to the conception of this paper, conceived the study design and drafted the manuscript. All authors read and approved the final version of the manuscript.

## Supplementary Material

Additional file 1 List of ICD-9-CM diagnosis codes for non-bacterial gastroenteritis, bacterial gastroenteritis, and dehydration. Click here for file

Additional file 2: Figure S1 Percentages of hospital admissions for gastroenteritis and dehydration by region (<3 months). *Note:* Gastroenteritis comprises both non-bacterial and bacterial diagnoses. *Data source:* Ministry of Health. Click here for file

Additional file 3: Figure S2 Percentages of hospital admissions for gastroenteritis and dehydration by region (3–11 months). *Note:* Gastroenteritis comprises both non-bacterial and bacterial diagnoses. *Data source:* Ministry of Health. Click here for file

## References

[B1] Wolfe I Thompson M Gill P Tamburlini G Blair M van den Bruel A Ehrich J Pettoello-Mantovani M Janson S Karanikolos M McKee M Health services for children in western Europe Lancet 2013 38 9873 1224 1234 23541056 10.1016/S0140-6736(12)62085-6

[B2] McDonald KM Approach to improving quality: the role of quality measurement and a case study of the agency for healthcare research and quality pediatric quality indicators Pediatr Clin N Am 2009 56 4 815 829 10.1016/j.pcl.2009.05.009 19660629

[B3] McDonald KM Davies SM Haberland CA Geppert JJ Ku A Romano PS Preliminary assessment of pediatric health care quality and patient safety in the United States using readily available administrative data Pediatrics 2008 122 2 e416 e425 10.1542/peds.2007-2477 18676529

[B4] Caminal J Starfield B Sánchez E Casanova C Morales M The role of primary care in preventing ambulatory care sensitive conditions Eur J Public Health 2004 14 3 246 251 10.1093/eurpub/14.3.246 15369028

[B5] Arah OA Westert GP Hurst J Klazinga NS A conceptual framework for the OECD health care quality indicators project Int J Qual Health Care 2006 18 Suppl 1 5 13 16954510 10.1093/intqhc/mzl024

[B6] AHRQ Measures of Pediatric Healthcare Quality Based on Hospital Administrative Data 2006 Rockville: The pediatric quality indicators

[B7] AHRQ pediatric quality indicators http://www.qualityindicators.ahrq.gov/Modules/pdi_resources.aspx

[B8] Programma Nazionale Valutazione Esiti http://95.110.213.190/PNEed13/index.php

[B9] Flores G Abreu M Chaisson CE Sun D Keeping children out of hospitals: parents’ and physicians’ perspectives on how pediatric hospitalizations for ambulatory care-sensitive conditions can be avoided Pediatrics 2003 112 5 1021 1030 10.1542/peds.112.5.1021 14595041

[B10] Albano F Lo Vecchio A Guarino A The applicability and efficacy of guidelines for the management of acute gastroenteritis in outpatient children: a field-randomized trial on primary care pediatricians J Pediatr 2010 156 2 226 230 10.1016/j.jpeds.2009.07.065 19836027

[B11] Lanata CF Fischer-Walker CL Olascoaga AC Torres CX Aryee MJ Black RE Child Health Epidemiology Reference Group of the World Health Organization and UNICEF Global causes of diarrheal disease mortality in children <5 years of age: a systematic review PLoS One 2013 8 9 e72788 10.1371/journal.pone.0072788 24023773 PMC3762858

[B12] Center for disease control and prevention http://www.cdc.gov/vaccines/vpd-vac/rotavirus

[B13] Curns AT Steiner C Barrett M Hunter K Wilson E Parashar UD Reduction in acute gastroenteritis hospitalizations among US children after introduction of rotavirus vaccine: analysis of hospital discharge data from 18 US States J Infect Dis 2010 201 11 1617 1624 10.1086/652403 20402596

[B14] Vitale F Barbieri M Dirodi B Vitali Rosati G Franco E A full economic evaluation of extensive vaccination against rotavirus with RIX4414 vaccine at National and Regional level in Italy Ann Ig 2013 25 1 43 56 23435779 10.7416/ai.2013.1905

[B15] Luciano L Lenzi J McDonald KM Rosa S Damiani G Corsello G Fantini MP Empirical analysis of the indicator ‘pediatric asthma hospitalization rate’ Ital J Pediatr 2014 40 1 7 10.1186/1824-7288-40-7 24447802 PMC3899920

