# Supplementary material for: Empirical examination of the indicator ‘pediatric gastroenteritis hospitalization rate’ based on administrative hospital data in Italy
Source: Ital J Pediatr. 2014 Feb 11;40:14. doi: 10.1186/1824-7288-40-14 (PMC3923239; doi:10.1186/1824-7288-40-14)
Supplement: Additional file 1 — List of ICD-9-CM diagnosis codes for non-bacterial gastroenteritis, bacterial gastroenteritis, and dehydration. [file 1824-7288-40-14-S1.pdf]

LIST OF DIAGNOSIS CODES INCLUDED IN THE STUDY

(i) A primary ICD-9-CM diagnosis code for non-bacterial gastroenteritis;

(ii) A primary ICD-9-CM diagnosis code for bacterial gastroenteritis;

(iii) A secondary ICD-9-CM diagnosis code for gastroenteritis (either bacterial or non-bacterial) and a primary ICD-9-CM diagnosis code for dehydration.

(i) ICD-9-CM Non-bacterial gastroenteritis diagnosis codes:

008.61 Enteritis due to rotavirus

008.62 Enteritis due to adenovirus

008.63 Enteritis due to Norwalk virus

008.64 Enteritis due to other small round viruses [SRV's]

008.65 Enteritis due to calcivirus

008.66 Enteritis due to astrovirus

008.67 Enteritis due to enterovirus NES

008.69 Other viral enteritis

008.8 Intestinal infection due to other organism, not elsewhere classified

009.0 Infectious colitis, enteritis, and gastroenteritis

009.1 Colitis, enteritis, and gastrointestinal of presumed infectious origin

009.2 Infectious diarrhea

009.3 Diarrhea of presumed infectious origin

558.9 Other and unspecified noninfectious gastroenteritis and colitis

(ii) ICD-9-CM Bacterial gastroenteritis diagnosis codes:

003.0 Salmonella gastroenteritis

003.8 Other specified salmonella infections

003.9 Salmonella infection, unspecified

004.0 Shigella dysenteriae

004.1 Shigella flexneri

004.2 Shigella boydii

004.3 Shigella sonnei

004.8 Other specified shigella infections

004.9 Shigellosis, unspecified

008.00 Intestinal infection due to E. coli, unspecified

008.01 Intestinal infection due to enteropathogenic E. coli

008.02 Intestinal infection due to enterotoxigenic E. coli

008.03 Intestinal infection due to enteroinvasive E. coli

008.04 Intestinal infection due to enterohemorrhagic E. coli

008.09 Intestinal infection due to other intestinal E. coli infections

008.1 Intestinal infection due to arizona group of paracolon bacilli

008.2 Intestinal infection due to aerobacter aerogenes

008.3 Intestinal infection due to proteus (mirabilis) (morganii)

008.41 Intestinal infection due to staphylococcus

008.42 Intestinal infection due to pseudomonas

008.43 Intestinal infection due to campylobacter

008.44 Intestinal infection due to yersinia enterocolitica

008.45 Intestinal infection due to Clostridium difficile

008.46 Intestinal infection due to other anaerobes

008.47 Intestinal infection due to other gram-negative bacteria

008.49 Intestinal infection due to other organisms

008.5 Bacterial enteritis, unspecified

(iii) ICD-9-CM Dehydration diagnosis codes:

276.5 Volume depletion disorder

276.50 Volume depletion, unspecified

276.51 Dehydration

276.52 Hypovolemia

Italicized code has become invalid in 2007 ICD-9-CM.
