# Supplementary figures and images for: Empirical examination of the indicator ‘pediatric gastroenteritis hospitalization rate’ based on administrative hospital data in Italy
Source: Ital J Pediatr. 2014 Feb 11;40:14. doi: 10.1186/1824-7288-40-14 (PMC3923239; doi:10.1186/1824-7288-40-14)

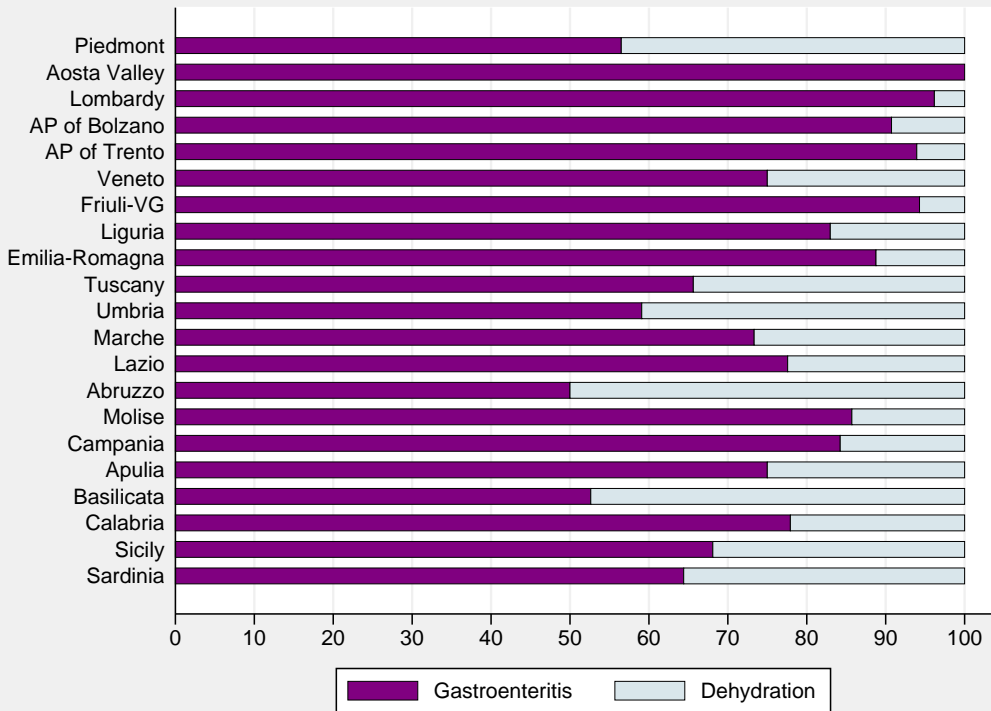

Supplement: Additional file 2: Figure S1 — Percentages of hospital admissions for gastroenteritis and dehydration by region (<3 months). Note: Gastroenteritis comprises both non-bacterial and bacterial diagnoses. Data source: Ministry of Health. [file 1824-7288-40-14-S2.pdf]

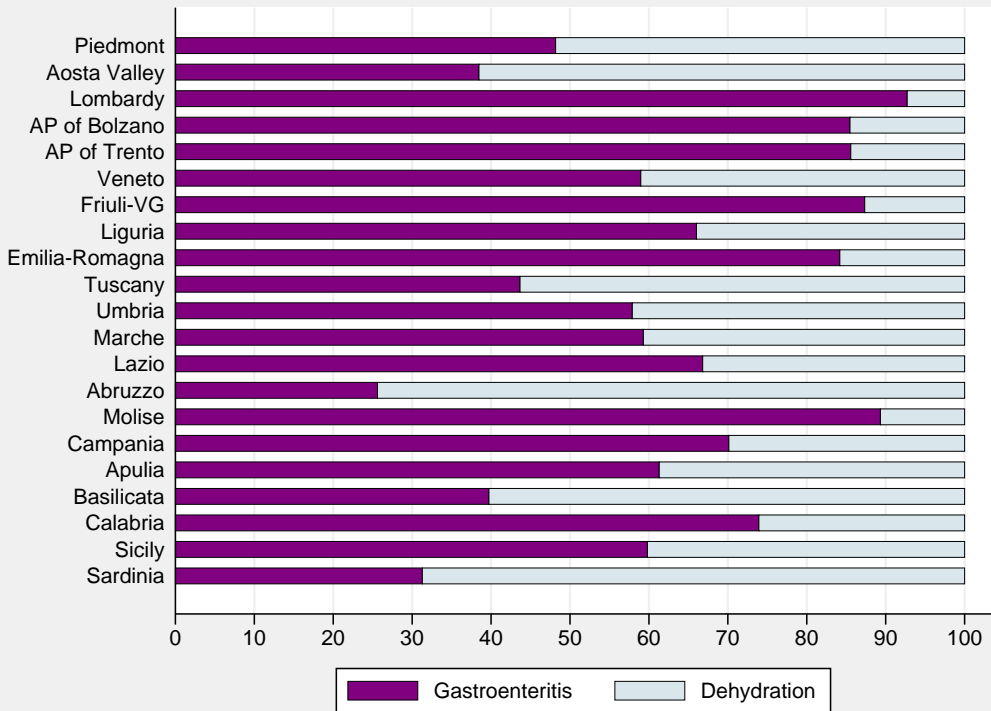

Supplement: Additional file 3: Figure S2 — Percentages of hospital admissions for gastroenteritis and dehydration by region (3–11 months). Note: Gastroenteritis comprises both non-bacterial and bacterial diagnoses. Data source: Ministry of Health. [file 1824-7288-40-14-S3.pdf]
